# De novo headache in ischemic stroke patients treated with thrombectomy: a prospective study

**DOI:** 10.1186/s10194-022-01455-3

**Published:** 2022-07-21

**Authors:** Daniel Gallo, Leire Manrique, Marcos Polanco, Andrés González-Mandly, Eduardo Torres, Enrique Palacio, José Luis Vázquez, Sara Pérez-Pereda, Vicente González-Quintanilla, Jorge Madera, Julio Pascual

**Affiliations:** Services of Neurology and Radiology, University Hospital Marqués de Valdecilla, Universidad de Cantabria and IDIVAL, Av. Valdecilla s/n, 39008 Santander, Cantabria Spain

**Keywords:** Endovascular thrombectomy, Headache, ICHD-3, Stroke

## Abstract

**Background and aim:**

Headache attributed to intracranial endovascular procedures is described in the ICHD-3. Our aim was to study the frequency and characteristics of headache specifically related to thrombectomy in patients with ischemic stroke.

**Methods:**

Prospective evaluation of clinical features of headache after thrombectomy using an ad hoc questionnaire.

**Results:**

One hundred seventeen patients were included (52.1% females). Most had an anterior circulation artery occlusion (91.5%). 93 (79.5%) received general anaesthesia. 111 (94.9%) required stent retriever, 21 (24.4%) angioplasty and 19 (16.2%) aspiration thrombectomy. 31 (26.5%; 95% CI 18.8–35.5%) had headache related to thrombectomy, and it was associated with a history of primary headache (*p* = 0.004). No differences about sex, initial NIHSS score, or the type or complexity of the procedure were observed. Headache was usually moderate and oppressive, ipsilateral to the artery occlusion and usually lasted less than 48 hours.

**Conclusions:**

Almost one-third of patients with ischemic stroke who undergo endovascular thrombectomy experience headache in the first 24 hours, occurring more frequently in patients who had a previous history of headaches regardless of the procedure complexity.

**Supplementary Information:**

The online version contains supplementary material available at 10.1186/s10194-022-01455-3.

## Highlights

- About one third of the patients who undergo a thrombectomy for acute stroke experience headache directly related to the procedure.

- Having a previous history of primary headache is associated with the presence of headache after thrombectomy.

- Headache related to thrombectomy usually coincides with the distribution of the affected artery, although it is bilateral in 45% of the cases, mostly oppressive, of an average duration between 1 and 2 days and of a moderate intensity.

**Supplementary Information:**

The online version contains supplementary material available at 10.1186/s10194-022-01455-3.

## Introduction

Headache caused directly by an intracranial endarterial procedure is described in the current Classification of Headache Disorders (ICHD-3) as “unilateral, ipsilateral to the procedure and lasting less than 24 hours” [1]. Its diagnostic criteria require at least 3 of the following 4: 1) headache has developed within 1 week of the procedure; 2) headache has resolved within 1 month after the procedure; 3) headache is ipsilateral to the procedure or bilateral; and 4) headache has one of the following sets of characteristics: a) severe, occurring abruptly within seconds of the procedure and lasting < 1 hour, b) moderate to severe, developing within hours of the procedure and lasting > 24 hours, and c) occurring in a patient with migraine and having the features of migraine with or without aura. These features define the three subtypes of headache attributed to an intracranial endarterial procedure recognized in the ICDH-3 classification. Mainly in terms of headache duration, current criteria are confusing, possibly as intracranial endarterial procedures today encompass a variety of procedures, for example, angioplasty, embolization, stent placement, etc., which depend on the indication to be treated [2–8].

Endovascular mechanical thrombectomy is the most efficacious treatment for ischemic stroke secondary to occlusion of brain arterial circulation [9–14]. Like most treatments, mechanical thrombectomy also has side effects. Some of the best known are arterial wall dissection or bleeding due to vessel perforation. However, a less serious, but not negligible, side effect could be the headache that this kind of interventional therapy can produce.

In this work we prospectively studied the frequency and characteristics of de novo headache related to thrombectomy in a series of consecutive patients attending our hospital with acute ischemic stroke.

## Methods

Our aim was to prospectively study de novo headaches in a minimum of 100 patients (with headache in a minimum of 30) in whom a mechanical thrombectomy due to ischemic stroke was performed. This was an observational, prospective study, with the beginning of data collection in November 2019 and completion in January 2021. In this period, all patients who underwent mechanical thrombectomy in our hospital were collected. This study had been approved by the Ethics Committee of Cantabria. We did not include those patients under 18 years of age, those whose anamnesis was not possible due to severe language impairment or decreased level of consciousness, those with arterial dissection or arterial rupture and those with headache on arrival to our hospital prior to thrombectomy.

The interview (an ad hoc questionnaire containing 32 items) took place at the end of the endovascular procedure, and by 24 hours of it. We collected data for demographic variables, medical antecedents, use of medications and, specifically, headache history before and after the ischemic episode and its main characteristics (location, quality, intensity, duration, need for analgesia). The data obtained on the ischemic event were the following: clinical-radiological diagnosis, the value of the ASPECTS interpretation (given by the radiologist), the clogged or stenosed artery and its affected segment and the NIHSS scale score, both, before and after endovascular treatment. Regarding the procedure itself, the following variables related to the intervention were also recorded: need for general anaesthesia, duration of the procedure in minutes, stent placement or not, and whether the thrombus was aspirated. All patients underwent at least a control CT brain scan 24 hours after mechanical thrombectomy.

A descriptive analysis was performed in which the qualitative variables are presented with their absolute (N) and relative (percentage) frequencies, and the quantitative variables are described by a central tendency measure (mean or median) and a dispersion measure [mean deviation (MD) or interquartile range (IQR)]. The distribution of quantitative variables was analysed using the Kolmogorov-Smirnov test. Only the variables “age” and “NIHSS at admission” for the headache and non-headache subgroups had a normal distribution. We used Student’s t-test to compare the continuous variables with both normal and non-normal distribution (given that *n* > 30), the Mann-Witney U test or Wilcoxon test to compare ordinal variables and the Chi2 test (or Fisher’s exact test when necessary) for the categorical variables. The analysis was performed with the statistical software SPSS. Significance was set at *p* < 0.05.

## Results

### Participants

**Fig. 1:**
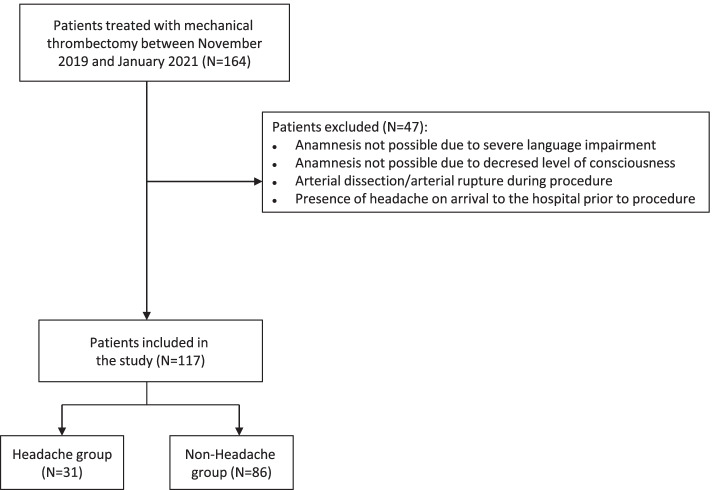
Flow chart with number of patients included and excluded in the study

During the study period 164 thrombectomies were performed in our hospital. As shown in Fig. 1, 47 patients were excluded. The study finally included a total of 117 patients of whom 61 (52.1%) were women, with a mean age of 68.8 years (MD 13.7; range 43 to 90). Thirty-one patients (26.5%; 95% CI 18.8–35.5%) had headache related to the procedure.

### Main medical antecedents

Vascular risk factors were highly prevalent: 68 (58%) had dyslipidaemia, 40 (34.2%) were smokers or ex-smokers, 68 (58.1%) suffered from arterial hypertension and 16 patients (13.7%) had type 2 diabetes mellitus. Fifty-three (45.3%) patients were taking angiotensin converting enzyme inhibitors (ACEI) or aldosterone 2 receptor antagonists (ARA2), 36 (30.8%) diuretics and 22 (18.8%) calcium antagonists. Forty-six (39.3%) patients were under statin treatment. Finally, 39 (33.3%) patients were on anticoagulant or antiplatelet treatment.

Fifteen (13%) patients reported to a previous history of headache. Five (33%) met migraine criteria and eight (53%) of tension-type headache. The remaining two had another type of headache according to ICHD-3 classification (one of them was a headache attributed to arterial hypertension, and the other was a headache attributed to Chiari malformation type 1).

### Characteristics of the ischemic episode

**Fig. 2:**
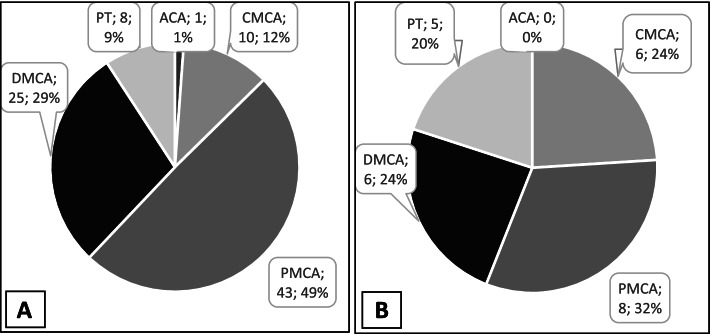
Distribution of CT scan ischemic lesion in all patients (**A**) and in patients who experienced headache (**B**). ACA: anterior cerebral artery, CMCA: complete middle cerebral artery, PMCA: partial middle cerebral artery, DMCA: deep middle cerebral artery, PT: posterior territory

A total of 107 (91.5%) patients suffered damage to the anterior circulation, while 10 (8.5%) had posterior circulation impairment. The specific diagnoses prior to thrombectomy are illustrated in Fig. 2. ASPECTS values median was 9 (IQR 2), while median NIHSS scale at admission and post-thrombectomy was 12 (IQR 10.5) and 4 (IQR 11.5), respectively (*p* = 0.000).

### Data related to the procedure

Twenty (17.1%) of the 117 patients included had been previously treated with iv alteplase. A total of 93 (79.5%) patients received general anaesthesia (median duration 29 minutes; IQR 42). Thrombectomy procedure was performed by using a stent retriever in 98 (83.8%), 6 only by aspiration (5,1%) and 13 by both (11.1%).Twenty-nine (24.8%) underwent thrombectomy plus angioplasty, of whom 20 underwent carotid stent and 1 intracranial stent implantation.

### Comparison of patients with headache vs patients without headache

There were no significant differences related to sex, mean age or the treatments previously received between the headache and non-headache subgroup.

Regarding the procedure itself, general anaesthesia was performed with a similar duration in both subgroups. Aspiration thrombectomy was conducted in 16% of patients in both subgroups. Mechanical thrombectomy with stent retriever was required in 28 (90%) of the patients who developed headache after the procedure vs 83 (97%) of the patients with no headache. Additionally, 8 (26%) patients of the headache subgroup needed carotid or intracranial angioplasty (5 with carotid stent implantation and none with intracranial stent implantation) vs 21 (24%) of the non-headache subgroup (15 with carotid stent and 1 with intracranial stent implantation). We did not find significant differences regarding the number of patients submitted to mechanical thrombectomy or angioplasty between both subgroups (*p* = 0.189 and *p* = 1.000 respectively, Fisher’s test), neither with number of attempts to recanalize (*p* = 0.246) or with recanalization rate (*p* = 0,818).

A control CT was performed 24 hours after thrombectomy in all patients. There were no significant differences between the number of patients with residual ischemic lesions in control CT in the headache vs non-headache groups (80.6% vs 71.8% *p* = 0.473, Fisher’s test). The rate of haemorrhagic transformation in the control CT scan was nearly the same in both subgroups. Analysing the relationship between headache and the resulting ischemic area, we found that headache was more frequent in those who had an area of established infarction in the entire middle cerebral artery (*p* = 0.021) although it was present in only 19% of them. Comparisons between headache and non-headache subgroups are listed in Table 1.

**Table 1:** Comparison of patients with headache vs patients without headache

	Headache group	Non-headache group	Test
**Age**	Mean 66.94 ± 12.81 years	Mean 69.50 ± 13.98 years	*p* = 0.373¶
**Sex**	19 (61%) women vs 12 (39%) men	42 (49%) women vs 44 (51%) men	*p* = 0.296*
**Headache’s history**	9 (29%)	6 (7%)	*p* = 0.004*
**NIHSS scale score prior to thrombectomy**	Mean 13.16 ± 7.03	Mean 13.02 ± 6.57	*p* = 0.998†
**ASPECTS score**	Mean 8.71 ± 1.55	Mean 9,07 ± 1.08	*p* = 0,467†
**Affected circulation**	Anterior 26 (84%) vs Posterior 5 (16%)	Anterior 81 (94%) vs Posterior 5 (6%)	*p* = 0.127*
**General anaesthesia**	26 (84%)	67 (78%)	*p* = 0.608*
**Duration of the procedure**	Mean 41.06 ± 30.79 min	Mean 42.20 ± 34.97 min	*p* = 0.874
**Residual lesion in control CT scan**	MCA:	MCA:	*p* = 0.064
	6 (5.1%) Complete middle cerebral territory	4 (3.4%) Complete middle cerebral territory	
	8 (6.8%) Partial middle cerebral territory	35 (29.9%) Partial middle cerebral territory	
	6 (5.1%) Deep middle cerebral territory	19 (16.2%) Deep middle cerebral territory	
	3 (10%) Posterior cerebral artery territory	1 (1%) posterior cerebral artery territory	*p* = 0.056*
	2 (6%) Basilar artery territory	2 (2%) basilar artery territory	*p* = 0.286*
	No ischemic lesion 6 (19%)	No ischemic lesion 24 (28%)	*p* = 0.347*
	Haemorrhagic transformation 7 (23%)	Haemorrhagic transformation 19 (22%)	*p* = 1.000*

¶ Student’s t test

†Mann-Witney’s U test

*Fisher’s exact test

#X^2^ test

### Post-mechanical thrombectomy headache characteristics

Although the majority of patients (71%) with post-thrombectomy headache had no prior history of headache, a relationship was found between having previous history of primary headache and the presence of headache after the procedure (30% vs 7% *p* = 0.004). The percentage of post-thrombectomy headache in the subgroup of those previously diagnosed with primary headache was 80% in migraineurs and 37.5% in tension-type headache patients.

Mean duration of posthrombectomy headache was 41.4 hours (median 24, range 24 to 168 hours) and it was self-limited in the first 24 h in 51.6% of the patients. Headache was bilateral in 13 (42%) patients and unilateral in 18 (58%): frontal in 10 (32%), parietal in 5 (16%), retroocular in 2 (7%) and occipital in 1 (3%).

Pain was most commonly referred as oppressive (19; 61.3%) and of moderate intensity (20; 64.5%). Twenty-six (83.9%) patients needed specific analgesia for their headaches. Regarding accompanying symptoms, 4 (12.9%) patients had nausea and/or vomiting, and 1 (3.2%), photophobia.

The location of the headache was usually associated with the site of the stroke: 75% of the patients with left hemicranial headache had a left hemispheric stroke (*p* = 0.032), 88.9% of patients with right hemicranial headache had a right hemispheric stroke (*p* = 0.015) and two of the fourteen patients (14.3%) who had a bilateral headache had a bilateral stroke (*p* = 0.576). According to the three subforms of headache attributed to an intracranial endarterial procedure described in the ICHD-3 classification, twenty-seven patients (87.1%) had a headache developing within hours to 1 day following the procedure. The remaining three patients (12.9%) had a headache meeting migraine criteria. There was no patient whose headache onset could be considered as abrupt. Main characteristics of post-mechanical thrombectomy headache are summarized in Table 2.

**Table 2:** Headache characteristics in headache related to thrombectomy

**Location:**	
- Holocranial 13 (42%)	
- Frontal 10 (32%)	
- Parietal 5 (16%)	
- Occipital 1 (3%)	
- Retroocular 2 (7%)	
**Laterality:**	
- Bilateral 14 (45%)	
- Unilateral 17 (55%)	
○ Left 8 (26%)	
○ Right 9 (29%)	
**Quality:**	
- Oppressive 19 (61%)	
- Pulsatile 12 (39%)	
**Intensity:**	
- Mild 3 (10%)	
- Moderate 20 (64%)	
- Severe 8 (26%)	
**Need of analgesia** 26 (84%)	
**Accompanying symptoms:**	
- Nausea or vomiting 4 (13%)	
- Photophobia or sonophobia 1 (3%)	
**Duration:**	
- 0–24 hours 16 (52%)	
- 24–48 hours 11 (35%)	
- 48–72 hours 3 (10%)	
> 72 hours 1 (3%)	

## Discussion

Endovascular mechanical thrombectomy has revolutionized the treatment of acute ischemic stroke [9–14]. Considering its current expansion, it seems necessary to define the prevalence and characteristics of headache in direct relationship with this specific procedure. The main finding of our work is that nearly one in three patients undergoing mechanical thrombectomy experiences de novo headache. Our data, therefore, confirm that a relevant number of patients undergoing this procedure will notice new onset headache.

To the best of our knowledge, there is only one study that has analysed specifically headache related to thrombectomy in a final sample of 96 patients [14]. The methodology of this study, which used the ICDH-3 beta version, was somewhat different as it was retrospective and focussed on “headache status before and after endovascular thrombectomy”, but not exactly on the acute headache related to the procedure. In fact, they concluded that there is a significant drop in migraine prevalence and an increase in tension-type headache prevalence after the mechanical thrombectomy (versus prior the procedure). The frequency of new onset headache in their work was only 11%, which is well explained by its retrospective data collection. Our results concur with those coming from the prospective Chinese study in 73 patients with unruptured intracranial aneurysms with endovascular treatment in whom new-onset headache was noticed in 24.1% of their patients [15].

One of our main objectives was to analyse the characteristics of this headache after the new ICHD-3 Classification [1]. In our experience, headache related to the procedure lasted typically 1–2 days (shorter than one week), was ipsilateral to the procedure or bilateral, usually oppressive, moderate and with a low rate of typical migraine accompanying symptoms, such as sono/photophobia or nausea/vomiting. Although, in general, headache directly related to thrombectomy meets the current ICHD-3 criteria for 6.7.1. Headache attributed to an intracranial endarterial procedure, the onset and duration criteria in its current version are misleading as explained above and fit much better with the subform “b) headache developing within hours to one day following the procedure and lasting a few days”. Our clinical observations can help in the future to refine the specific diagnostic criteria of headache attributed to intracranial endarterial procedures and, specifically, of headache related to mechanical thrombectomy.

Our work has several limitations. Although we found a relationship between performing mechanical thrombectomy and the appearance of headache, and even taking into account that we excluded patients with headache prior to the procedure, it is still possible that in some cases headache could be due to the stroke or angioplasty itself or to factors such as vasospasm, dissection or medications [16–19]. In our study almost one-third of patients experienced de novo headache related to the procedure, although it must be taken into account that general anaesthesia was used in a high number of patients and we could have missed some headaches because of this. Even though the relatively low numbers of patients experiencing headaches explain the lack of statistical significance of many variables in this series, numerical data indicate that referring to a history of primary headache could be a risk factor for the development of headache related to thrombectomy. Procedural complexity did not increase the proportion of headache. The real value of these clinical variables as potentially predictors of incidence and severity of headache related to thrombectomy should be validated in future studies with a higher number of patients.

## Conclusions

This prospective study shows that almost one out of three patients with ischemic stroke who undergo a thrombectomy will experience headache in the first 24 hours usually oppressive, moderate and lasting an average of 1–2 days. Headache characteristics meet well current ICHD-3 criteria for the subtype b of 6.7.1 Headache attributed to an intracranial procedure.

## Supplementary Information

**Additional file 1.**

**Additional file 2.**

**Additional file 3.**

## Data Availability

Data from this study are available on request to the corresponding author.
