# Supplementary material for: De novo headache in ischemic stroke patients treated with thrombectomy: a prospective study
Source: J Headache Pain. 2022 Jul 21;23(1):85. doi: 10.1186/s10194-022-01455-3 (PMC9306161; doi:10.1186/s10194-022-01455-3)
Supplement: Supplementary file 1 — Additional file 1. [file 10194_2022_1455_MOESM1_ESM.docx]

| Número de Historia |  | | | | | | |
| --- | --- | --- | --- | --- | --- | --- | --- |
| Edad |  | | | | | | |
| Sexo | Hombre | | Mujer | | | | |
| Antecedentes Personales |  | | | | | | |
| Medicación Previa |  | | | | | | |
| Diagnóstico que lleva a la trombectomía |  | | | | | | |
| ASPECTS |  | | | | | | |
| Arteria obstruida/estenosada |  | | | | | | |
| Puntuación NIH previa trombectomía |  | | | | | | |
| Fibrinólisis previa trombectomía | Sí | | No | | | | |
| Cefalea previa trombectomía | Sí | | No | | | | |
| En caso de cefalea | Localización | | |  | | | |
|  | Calidad | | |  | | | |
|  | Intensidad | | |  | | | |
|  | Duración | | |  | | | |
|  | Necesidad de analgesia | | Sí | | | | No |
| Anestesia general | Sí | | No | | | | |
| Duración procedimiento |  | | | | | | |
| Colocación Stent | Sí | | No | | | | |
| Aspiración trombo | Sí | | No | | | | |
| Medicación durante procedimiento |  | | | | | | |
| NIH post-trombectomía |  | | | | | | |
| Cefalea durante o 24 h tras trombectomía | Sí | | No | | | | |
| Si cefalea | Localización | |  | | | | |
|  | Calidad | |  | | | | |
|  | Intensidad | |  | | | | |
|  | Duración | |  | | | | |
|  | Analgesia | Sí | | | | No | |
|  | Síntomas acompañantes | |  | | | | |
| Resultados TAC control 24 h NORMAL |  | | | | | | |
| Resultados TAC control 24 h ANORMAL | Infarto carótida | |  | | | | |
|  | Infarto ACM | |  | | | | |
|  | Infarto ACA | |  | | | | |
|  | Infarto ACP | |  | | | | |
|  | Infarto cortical ACM | |  | | | | |
|  | Infarto ramas profundas ACM (lenticulo-estriadas) | |  | | | | |
|  | Infarto lacunar | |  | | | | |
|  | Infarto troncoencefálico | |  | | | | |
|  | Signos de sangrado | | Sí | | | | No |
|  |  |  | Subaracnoidea | | | |  |
|  |  |  | Local | | Sí | No |  |

**HOJA DE RECOGIDA DE DATOS CEFALEA EN RELACIÓN CON TROMBECTOMÍA**
