# Supplementary material for: De novo headache in ischemic stroke patients treated with thrombectomy: a prospective study
Source: J Headache Pain. 2022 Jul 21;23(1):85. doi: 10.1186/s10194-022-01455-3 (PMC9306161; doi:10.1186/s10194-022-01455-3)
Supplement: Supplementary file 2 — Additional file 2. [file 10194_2022_1455_MOESM2_ESM.docx]

| Patient identification |  | | | | | | |
| --- | --- | --- | --- | --- | --- | --- | --- |
| Age |  | | | | | | |
| Sex | Male | | Female | | | | |
| Medical antecedents |  | | | | | | |
| Medication |  | | | | | | |
| Diagnosis before thrombectomy |  | | | | | | |
| ASPECTS |  | | | | | | |
| Affected Artery |  | | | | | | |
| NIHSS score pre-thrombectomy |  | | | | | | |
| Fibrinolysis | Yes | | No | | | | |
| Headache pre-thrombectomy | Yes | | No | | | | |
| If headache | Localization | | |  | | | |
|  | Quality | | |  | | | |
|  | Intensity | | |  | | | |
|  | Duration | | |  | | | |
|  | Analgesia | | Yes | | | | No |
| General anaesthesia | Yes | | No | | | | |
| Procedure duration |  | | | | | | |
| Stent | Yes | | No | | | | |
| Aspiration | Yes | | No | | | | |
| Treatments given during the procedure |  | | | | | | |
| NIHSS post-thrombectomy |  | | | | | | |
| Headache during thrombectomy or 24 h after | Yes | | No | | | | |
| If headache | Localization | |  | | | | |
|  | Quality | |  | | | | |
|  | Intensity | |  | | | | |
|  | Duration | |  | | | | |
|  | Analgesia | Yes | | | | No | |
|  | Accompanying symptoms | |  | | | | |
| CT after 24 h without abnormalities |  | | | | | | |
| Abnormal CT after 24 h | Carotid stroke | |  | | | | |
|  | CMA stroke | |  | | | | |
|  | CAA stroke | |  | | | | |
|  | PCA stroke | |  | | | | |
|  | CMA cortical stroke | |  | | | | |
|  | CMA stroke of deep branches | |  | | | | |
|  | Lacunar stroke | |  | | | | |
|  | Brainstem stroke | |  | | | | |
|  | Signs of bleeding | | Yes | | | | No |
|  |  |  | Subarachnoid | | | |  |
|  |  |  | Local | | Yes | No |  |

**DATA COLLECTION SHEET**
