# Supplementary material for: De novo headache in ischemic stroke patients treated with thrombectomy: a prospective study
Source: J Headache Pain. 2022 Jul 21;23(1):85. doi: 10.1186/s10194-022-01455-3 (PMC9306161; doi:10.1186/s10194-022-01455-3)
Supplement: Supplementary file 3 — Additional file 3. [file 10194_2022_1455_MOESM3_ESM.docx]

**Supplemental material**

Types of stents used in mechanical thrombectomy procedure.

| **Type of stent** | **Frequency** | **Percentage** |
| --- | --- | --- |
| 3 MAX | 5 | 4.3% |
| Catch mini | 9 | 7.7% |
| Catch mini + Preset | 1 | 0.9% |
| Catch mini + Rebar + Solitare | 1 | 0.9% |
| Catch mini + Solitare | 1 | 0.9% |
| Catch mini + Stentriever + Trevo | 1 | 0.9% |
| Embotrap | 15 | 12.8% |
| Embotrap + NeuroSlider | 1 | 0.9% |
| Embotrap + pRESET | 4 | 3.4% |
| Embotrap + Rebar | 6 | 5.1% |
| Jet7 | 1 | 0.9% |
| NeuroSlider + pRESET | 1 | 0.9% |
| NeuroSlider + Rebar | 1 | 0.9% |
| Neurospeed | 1 | 0.9% |
| pRESET | 10 | 8.5% |
| pRESET + Rebar | 6 | 5.1% |
| pRESET + Trevo | 2 | 1.7% |
| Progreat | 1 | 0.9% |
| Rebar | 11 | 9.4% |
| Rebar + Solitare | 13 | 11.1% |
| Solitare | 19 | 16.2% |
| Tigertriver | 1 | 0.9% |
| Trevo | 4 | 3.4% |
| Trevo XP | 1 | 0.9% |
| Trevo + pRESET | 1 | 0.9% |

Number of attempts to recanalize:

| **Number of attempts** | **Headache group** | | **Non-headache group** | |
| --- | --- | --- | --- | --- |
|  | **N (%)** | **Cumulative N (%)** | **N (%)** | **Cumulative N (%)** |
| 1 | 16 (51.6%) | 16 (51.6%) | 40 (46.5%) | 40 (46.5%) |
| 2 | 8 (25.8%) | 24 (77.4%) | 21 (24.4%) | 61 (70.9%) |
| 3 | 0 | 24 (77.4%) | 14 (16.3%) | 75 (87.2%) |
| 4 | 7 (22.6%) | 31 (100%) | 4 (4.6%) | 79 (91.8%) |
| 5 | 0 | 31 (100%) | 2 (2.3%) | 81 (94.1%) |
| 6 | 0 | 31 (100%) | 3 (3.5%) | 84 (97.6%) |
| 7 | 0 | 31 (100%) | 0 | 84 (97.6%) |
| 8 | 0 | 31 (100%) | 1 (1.2%) | 85 (98.8%) |
| 9 | 0 | 31 (100%) | 1 (1.2%) | 86 (100%) |

Recanalization analysis:

|  |  | **Headache group** | **Non-headache group** |  |
| --- | --- | --- | --- | --- |
| **TICI** | 0 | 0 | 4 | 4 |
|  | 2a | 1 | 3 | 4 |
|  | 2b | 4 | 11 | 15 |
|  | 2c | 5 | 14 | 19 |
|  | 3 | 20 | 51 | 71 |
|  | Total | 30 | 83 | 113 |

*X^2^ test: 1.547 p=0.818*
